# Standard Reference Materials for Cement Paste: Part II-Determination of Mixing Ratios

**DOI:** 10.3390/ma11050861

**Published:** 2018-05-22

**Authors:** Dong Kyu Lee, Myoung Sung Choi

**Affiliations:** Department of Safety Engineering, Dongguk University-Gyeongju, 123 Dongdae-ro, Gyeongju, Gyeongbuk 38066, Korea; dklee@dongguk.ac.kr

**Keywords:** flowability, standard reference materials, rheology, yield stress, plastic viscosity

## Abstract

A variety of special concrete structures have been designed for domestic and overseas construction markets that require highly advanced construction technology. Therefore, it is necessary to secure quantitative construction technology and develop a standard reference material (hereinafter: SRM) with consistent flow performance and quality in order to evaluate the quantitative performance of flowability. On the other hand, the flowability of concrete is influenced greatly by the flowability of the cement paste. In addition, considering the design strength and workability, the mix design was carried out at various mixing ratios, according to the purpose of the site. Therefore, based on the derived components of standard reference materials for cement paste, this paper proposes a mixing ratio for standard reference materials that can uniformly simulate the flow characteristics of cement paste, according to the water–cement ratio (*W*/*C*). The results show that yield stress was determined by the ratio of water and glycerol while plastic viscosity was controlled by the limestone content. Finally, the mixing ratio of standard reference materials that can simulate the rheological properties of cement paste by *W*/*C* was suggested.

## 1. Introduction

The construction industry has recently made remarkable progress in construction technology along with design and material technologies. In particular, construction technology has gained global recognition for its robust performance, which is demonstrated by mega-scale construction projects such as several high-rise building projects and its growing share in the global construction market. Special concrete construction has also become increasingly important throughout the construction industry with the rising demand for various types of special concrete structures in both domestic and overseas construction markets [1]. To ensure flawless construction of special concrete structures, it is essential to secure efficient concrete delivery and placement in addition to optimized concrete mixing designs [2]. This requires the objective analysis of design, construction, and quantitative evaluation techniques to enable optimal quality assurance and construction management [3]. In order to quantitatively evaluate the flow behavior of concrete, various studies have been conducted on the influence of aggregate size and surface condition [4,5], porosity [6], and volume fraction [7] of concrete. However, concrete is composed of particles of various sizes that range from several tens of μm to millimeters, which causes interactions between particles at the boundary of particles [8,9] as well as chemical reactions [10] and thixotropic behavior [11] that result in irregular flow characteristics [10]. Therefore, to secure more economical and effective construction technologies and to develop an efficient construction performance evaluation system, there is a growing need for the development of SRMs that can be used as the basis for quantitative flowability evaluation to ensure uniform quality management and performance evaluation. The development of SRMs that can reproduce the flowability of fresh concrete is essential for stable construction performance because they enable reliable quality control in the initial construction stages. SRMs provide absolute values for effective performance evaluation instead of relying on comparison-based relative values as seen in existing empirical tests [4]. The quantitative evaluation results can be used to make predictions in the pre-construction stage and conduct more accurate construction performance evaluation. Furthermore, SRMs are indispensable for the calibration of rheometers currently in use for a range of quantitative rheological measurement systems. They can also be used in a variety of fields where quantitative control of rheological behavior is required such as pipe hydrostatic circuit testing, pumping equipment abrasion evaluation test, and 3D digital printing. Therefore, although there have been a few studies about the development of standard reference materials for cementitious materials [12] and the study of materials that can accurately reproduce the dynamic characteristics of cement particles with non-hydratable properties [13,14,15]. There have been few studies on the performance evaluation of the standard reference materials.

In general, concrete can be divided into aggregates and cement paste and the flowability of fresh concrete is determined primarily by the rheological properties of fresh cement paste. Among the large variety of mix designs, an optimal mix design is determined according to the purpose of each site such as the type and use of the structure, which takes into account the compressive strength and workability. This suggests that the development of SRMs for accurate simulation of concrete rheological properties should be preceded by the development of SRMs for cement paste, which is the most basic constituent of concrete. According to the results obtained for SRM constituent materials for cement paste in a previous paper [16], the mixing ratio plays a significant role in developing an SRM for the flow model of each mixing ratio of cement paste, which takes the selected concrete mix design into account.

Therefore, in this study, based on the concept of rheology, which can evaluate the initial flow characteristics of cementitious materials by incorporating the results of the previous consecutive paper “Part I. Suggestion of Constituent Materials Based on Rheological Analysis” [16], the mixing ratio of standard reference materials can simulate the initial flow characteristics of cement paste by different *W*/*C*.

## 2. Experimental Design and Methods

### 2.1. Experimental Design

The requirements for particulate-phase SRM presented in the research are as follows [12]: (1) the SRM should resist particle separation during the test; (2) show a linear Bingham-type reaction in the broad-range stress–shear rate relationship; (3) resist changes caused by rheological and chemical interactions between the fluid and particles over a prolonged period of time; (4) have sufficient yield stress to prevent the separation of aggregates; and (5) should not exhibit nonlinear hysteretic behavior.

Drawing on the results from a previous study that determined the material composition satisfying all these requirements for particulate-phase SRM, this study aims to present the mixing ratios of SRMs for cement paste for different mix designs. The first experiment analyzed the rheological properties of cement pastes at different *W*/*C* ratios. The next step is to analyze the influence of SRM components on the overall rheology. Mixing ratios are proposed for the SRMs selected for the cement paste model based on the analysis results. Subsequently, each of the proposed SRM mixing ratios was tested to determine whether their performance satisfies the requirements for particulate-phase SRM.

**Figure 1:**
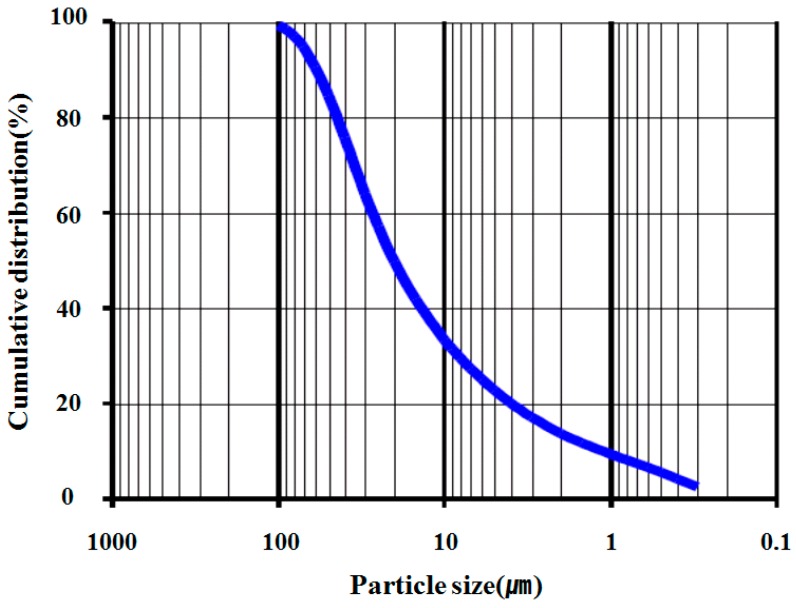
Particle size distribute (Limestone powder).

The components of the SRM for cement paste determined in a previous study are limestone as a cement substitute, glycerol as a matrix fluid substitute, and distilled water [16]. The type of cement used for the experiments was ordinary Portland cement. Table 1 and Table 2 show the compositional analysis results for the constituents of each material. In the case of glycerol, it was analyzed by a wet method and limestone was detected by X-ray fluorescence analysis Particle Size Distribution for limestone using Malven 3000 (Omya, Seoul, Korea) was also performed (see Figure 1).

### 2.2. Experimental Methods

**Figure 2:**
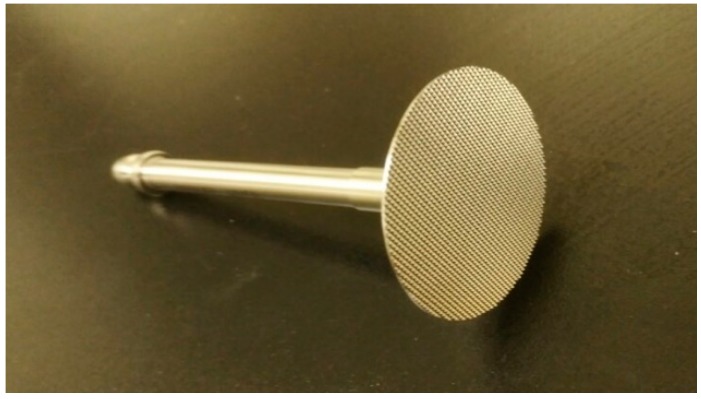
Picture of spindle.

A rheometer was used to evaluate quantitatively the rheological properties of the cement paste and SRMs. Rheological measurements are usually performed by determining the relationship between the shear stress acting on the material and the resulting shear strain. The Bingham model (see Equation (1)) [17,18,19,20,21,22] was used to determine the plastic viscosity and yield stress. Based on previous studies [17,18,19,20,21,22], most authors indicated that a linear relationship for the rheological analysis of cement paste is appropriate to determine rheology parameters. The plastic viscosity is defined as the slope of the shear stress vs. the shear rate curve and the yield stress is the value of the y-axis intersection point obtained by regression analysis [23,24]. The materials for the rheological measurements were mixed with a high-speed mixer for 120 s at four time-steps (15, 15, 30, and 60 s) by kneading the mixture after each step to ensure homogeneous mechanical properties. Prior to the measurement experiment, each specimen was subjected to pre-rotation (50 s^−1^/60 s) under constant temperature (20 °C) and time conditions to further homogenize the component materials, which is followed by a 10 s of rest to control the directionality of the component materials. At each measurement, the shear resistance against the rheometer spindle was measured at 10-step rotational rates predetermined along the upward and downward curves. The shearing rate increased from 0.1 to 40 s^−1^ and then decreased back to 0.1 s^−1^. The hysteresis is directly related to the experimental duration of the measuring cycle. Therefore, the experiment was conducted with a minimum time interval [25]. In addition, a serrated spindle with a diameter of 50 mm was used and the gap was determined to be 0.5 mm (see Figure 2) by considering that the slip phenomenon is affected by the gap between the parallel plate and the shearing surface and the spindle surface [26].

(1) $$\tau =\eta \dot{\gamma }+\tau_{0}$$

where τ, η, $\dot{\gamma }$, and $\tau_{0}$ are the shear stress, plastic viscosity, shear rate, and yield stress, respectively.

## 3. Analysis of the Proposed Mixing Ratios

### 3.1. Rheological Analysis of the Cement Paste

**Figure 3:**
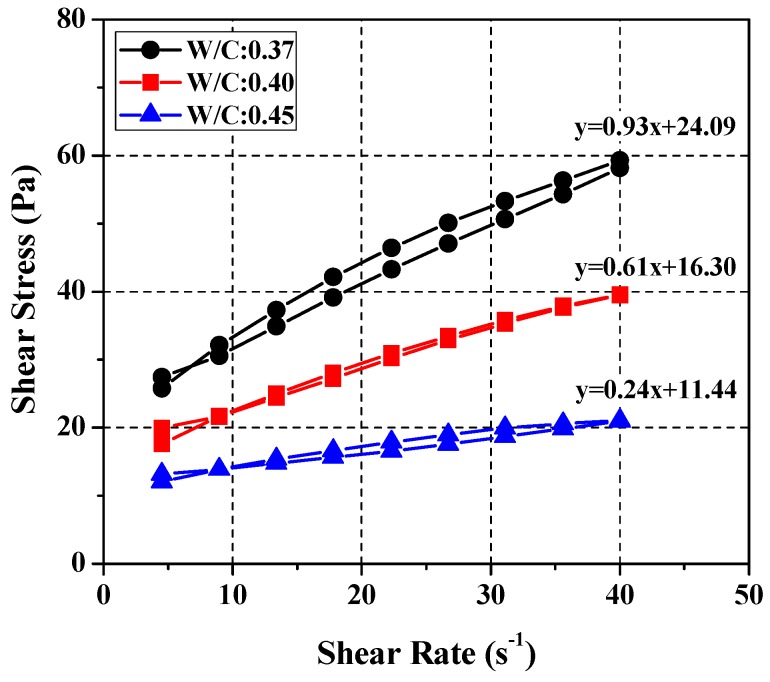
Test results of cement paste rheology.

To derive the yield stress and plastic viscosity of each cement paste mix the design for the SRM. Rheological analysis of the cement paste mixtures was first performed. Three mixing ratios (*W*/*C* = 0.37, 0.40, and 0.45) were analyzed experimentally by taking into account the common concrete mixing ratios and the tests were repeated several times under the condition of five-minute intervals after mixing to reflect the hydration-induced rheological weakening and multidimensional properties of the cement paste [27,28,29,30,31,32,33]. Figure 3 plots the rheological analysis results for the three cement paste mixtures. The results show that the tested cement pastes have somewhat low plastic viscosity and high yield stress values that decrease linearly with increasing *W*/*C*. Table 3 lists the means and ranges of the yield stress and plastic viscosity for each mixture of cement paste calculated using data from multiple experiments.

### 3.2. Developed Standard Reference Materials

**Figure 4:**
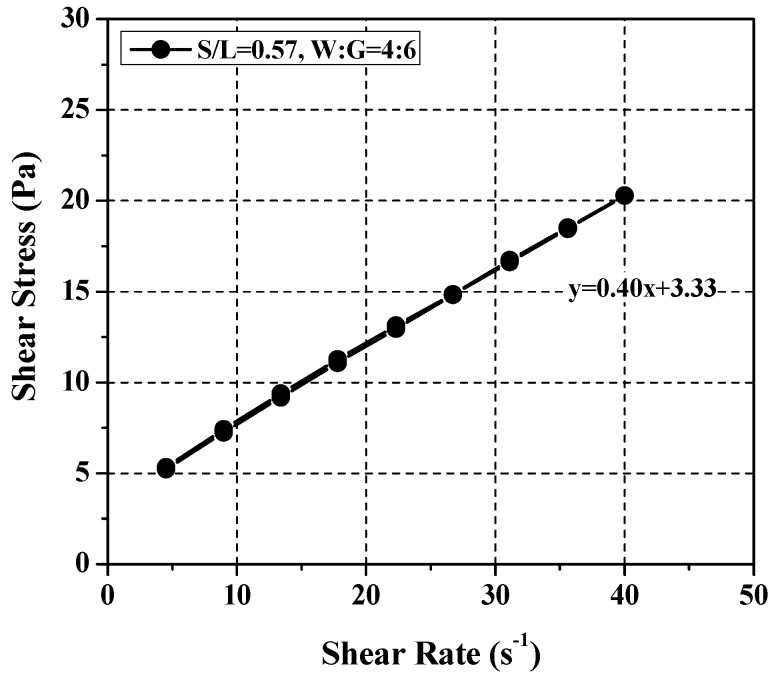
Test results of rheology for the initial condition.

**Figure 5:**
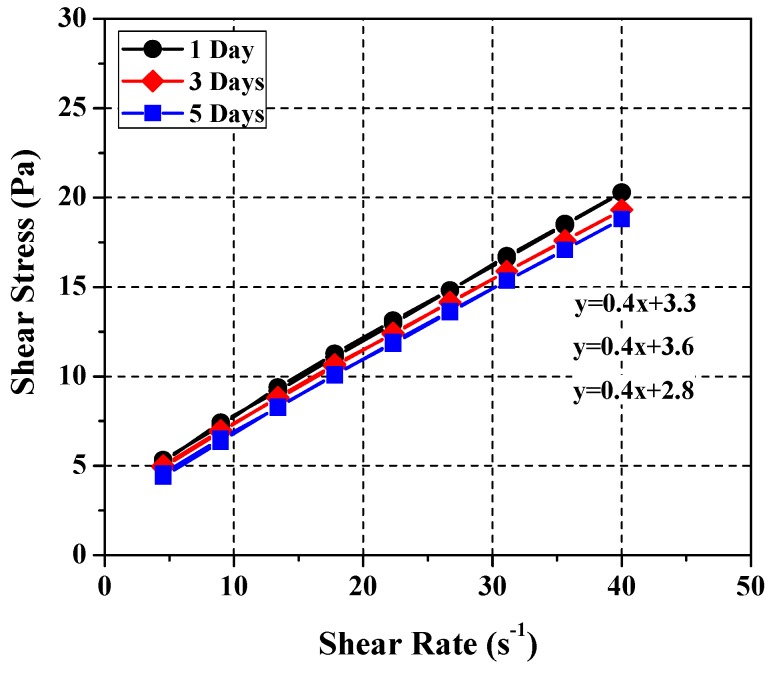
Test results of rheology for ages.

The experimentally-derived SRMs for cement paste consist of different combinations of limestone, glycerol, and distilled water [16]. According to Figure 4, they satisfy four out of the five requirements for the rheological properties of freshly mixed particulate-phase SRM. These four requirements are that it resists particle separation during testing, exhibits linear Bingham-type reaction in the broad-range shear stress–shear rate relationship, has sufficient yield stress to prevent the separation of aggregates, and does not show nonlinear hysteretic behavior. To verify if they meet all requirements, it should resist the changes caused by rheological and chemical interactions between the fluid and particles over a prolonged period of time. The age-dependent rheological properties was analyzed, which was shown in Figure 5. It was found that all the proposed SRMs showed satisfactory performance. In other words, it was confirmed that the derived standard reference components have constant rheological properties irrespective of the passage of time.

**Figure 6:**
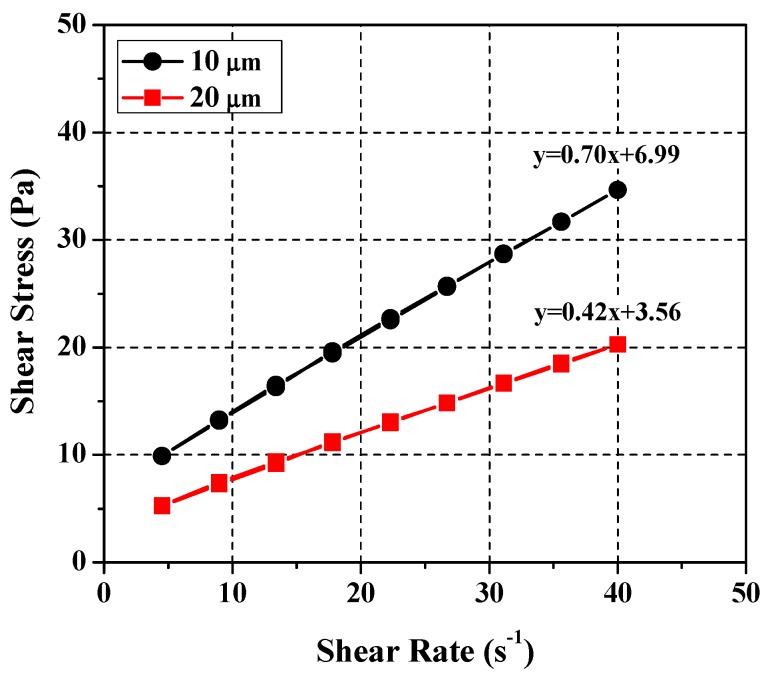
Test results of rheology for particle sizes.

Figure 6 presents the results of the rheological analysis performed using limestone particle size as the independent variable (10 μm, 20 μm). Although higher plastic viscosity and yield stress values were obtained with the 10 μm particle because of increased inter-particle contact friction. All the requirements for particulate-phase SRM were met by both particle sizes [27]. Considering the mean particle size of the cement paste, the limestone particle size was set to 20 μm. The basic mixing ratio was set to (glycerol + water)/limestone (hereinafter *S*/*L*) = 0.57 at water:glycerol = 4:6, which takes the mixing ratios presented in Ferraris et al. (2012) into consideration. The rheological analysis resulted in plastic viscosity and yield stress of 0.4 Pa·s and 3.3 Pa, respectively, which was plotted in Figure 4. The yield stress was lower than that of cement paste. In other words, a higher yield stress is required to derive rheological properties similar to those of cement paste. To determine the required level of yield stress, the influence of SRM components on the overall rheological properties was examined.

### 3.3. Factors Influencing the Rheological Properties of Each SRM Component

#### 3.3.1. Analysis of the Yield Stress

**Figure 7:**
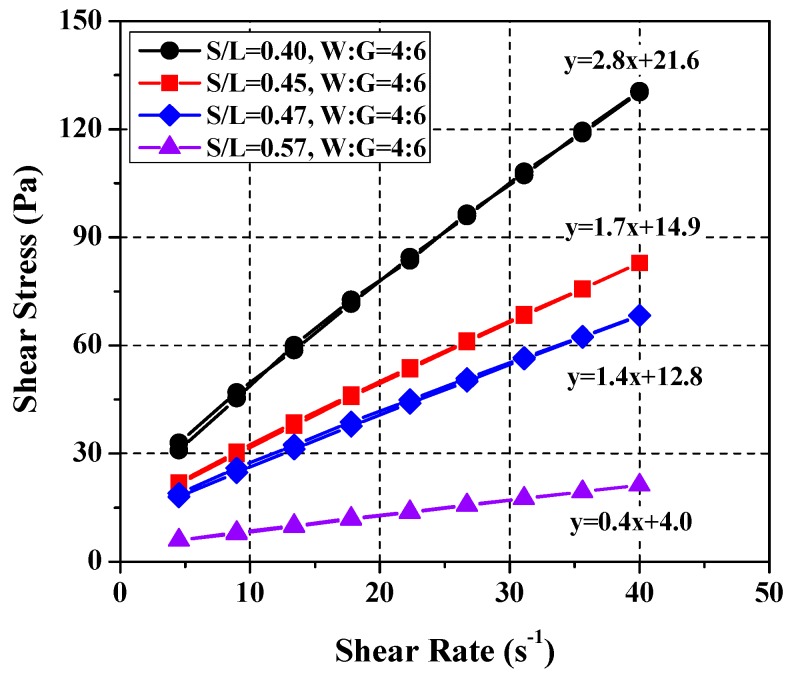
Variation of rheology depending on limestone ratios.

First the SRM mixing ratio that satisfies the cement paste mix of *W*/*C* = 0.37, which demonstrated the highest plastic viscosity and yield stress values in the rheological analysis, was determined. This served as the basis for deriving the mixing ratios corresponding to *W*/*C* = 0.40 and *W*/*C* = 0.45. As mentioned above, the cement paste mixing ratio of *W*/*C* = 0.37 had yield stress of 22 ± 3 Pa. Given the general tendency of the rheological properties of cement paste, i.e., the larger the cement proportion, the higher the plastic viscosity and yield stress analysis (see Figure 3) as well as rheological analysis was performed by varying the proportion of limestone at four levels (*S*/*L* = 0.40, 0.45, 0.47, and 0.57). The water–glycerol ratio was fixed at 4:6 in order to derive the SRM mixing ratio that satisfies the yield stress 22 ± 3 Pa. The mixing ratio of *S*/*L* = 0.40 in water was found to satisfy the yield stress. The experiment revealed a similar tendency for the cement paste. The plastic viscosity and yield stress increased linearly concomitantly with limestone proportion (see Figure 7). In other words, as the concentration of limestone increases, the concentration of the standard reference materials increases and the yield value and the plastic viscosity became higher since the concentration of the material including particles increased [26,27]. The yield stress, in particular, increased by a greater magnitude than the plastic viscosity, which suggests that the yield stress is influenced by limestone proportion in SRM.

#### 3.3.2. Analysis of Plastic Viscosity

The yield stress analysis showed that the mixing ratio of *S*/*L* = 0.40 with water:glycerol = 4:6 satisfies the cement paste mixing ratio of 22 ± 3 Pa at *W*/*C* = 0.37. In the case of the plastic viscosity, however, the SRM plastic viscosity (approximately 2.8 Pa·s) was slightly higher than that of cement paste at *W*/*C* = 0.37. Therefore, an experiment was conducted to reduce the plastic viscosity at a fixed yield stress. The experiment was performed at four water–glycerol ratios (4:6, 5:5, 6:4, and 7:3). The proportion of glycerol was reduced to the lowest possible level at which no hysteresis occurs. An earlier study showed that glycerol helps prevent material separation and hysteresis behavior [16].

**Figure 8:**
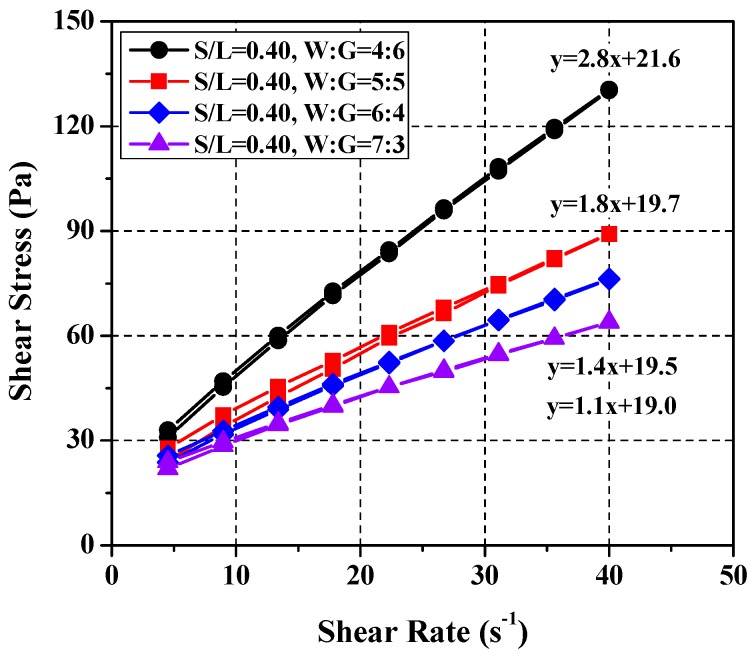
Variation of rheology depending on water and glycerol ratios.

As illustrated in Figure 8, plastic viscosity decreased with an increasing water–glycerol ratio at a constant yield stress (22 ± 3 Pa). This suggests that plastic viscosity decreases with an increasing proportion of water at the same *S*/*L* condition and does not significantly influence yield stress. Plastic viscosity was influenced greatly by the proportion of glycerol and water in the SRM rheology. In addition, an SRM mixing ratio of water:glycerol = 7:3 at *S*/*L* = 0.40 was found to satisfy the rheological properties of the cement paste mixing ratio of *W*/*C* = 0.37.

#### 3.3.3. Proposal of Mixing Ratios

An examination of the influence of SRM composition on rheological properties showed that the yield stress and plastic viscosity were influenced greatly by limestone content and proportion of glycerol, respectively. Furthermore, the SRM glycerol content of the mixing ratio that satisfies the rheological properties of the cement paste mixing ratio of *W*/*C* = 0.37 (i.e., water:glycerol = 7:3 and *S*/*L* = 0.40) was approximately 12% of the limestone content. The analysis results are expressed mathematically below.

(2) $$\frac{S}{L}=\frac{W+G}{L}=\frac{W}{L}+\frac{G}{L}$$

where the ratio of (*W* + *G*)/*L* influences the plastic viscosity. *L* influences the yield stress. *W*/*L* influences the plastic viscosity and yield stress. *G*/*L* affects on prevention of hysteresis behavior.

**Figure 9:**
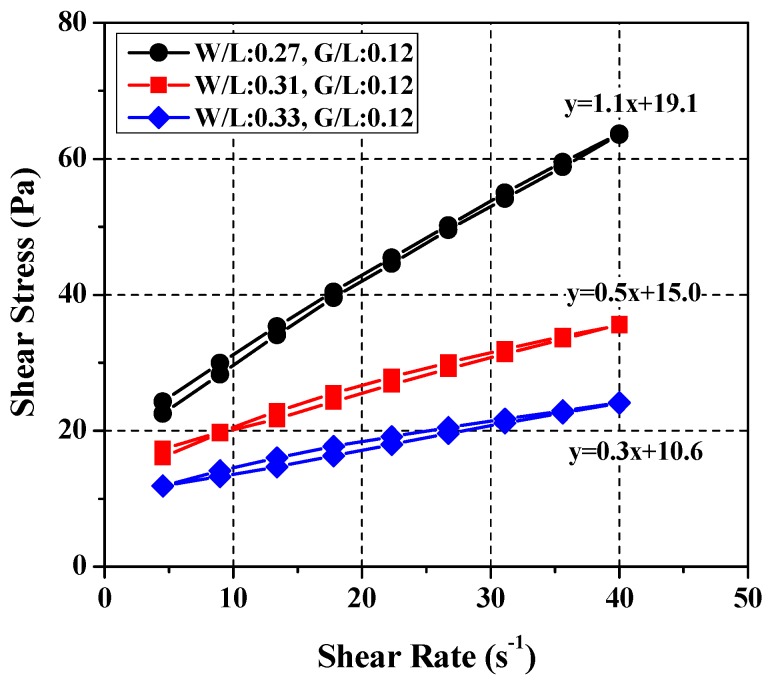
Test results of rheology for derived SRM mixing ratios.

Based on these results, experiments were performed at three water–limestone ratios (*W*/*L* = 0.27, 0.31, and 0.33) with glycerol content fixed at 12% of limestone content. The results are presented in Figure 9 and Table 4. The rheological value of cement paste with *W*/*C* = 0.37 is almost the same as that of SRM with *W*/*L* = 0.27 and *W*/*L* = 0.31 when *W*/*C* = 0.4 and *W*/*L* = 0.33 and when *W*/*C* = 0.45. In other words, all values satisfied the yield stress and plastic viscosity ranges of the cement paste obtained from the rheological analysis of each mix.

#### 3.3.4. Review of the SRM Requirements According to the Mixing Ratio

This study examined whether SRMs derived for each mixing ratio demonstrated that rheological properties satisfy the requirements for particulate-phase SRMs. As shown in Table 5, the derived SRMs satisfied all the requirements. This allowed us to determine which SRM mixing ratios are capable of simulating the selected cement paste mixing ratios, which was listed in Table 6, and establish Equation (3), which expresses the relationship between the mix designs of the cement paste and SRM.

(3) $$W/L=a{\left(W/C\right)}^{2}+b(W/C)+c$$

(4) G/L=0.12

where a, b, and c are 2.629, −1.491, and 0.471, respectively.

## 4. Validation of Suggested Mixing Ratio for SRM

**Figure 10:**
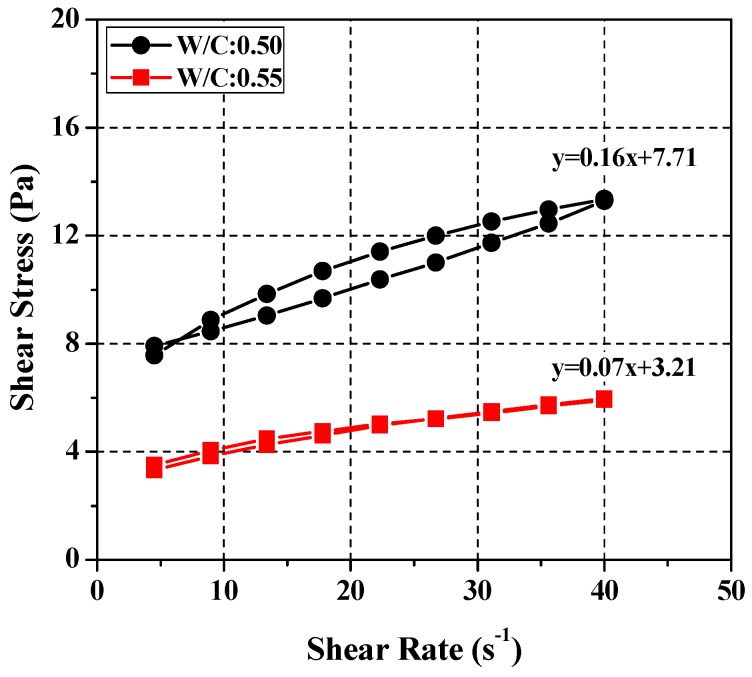
Test results of rheology for cement paste (*W*/*C* = 0.50, 0.55).

**Figure 11:**
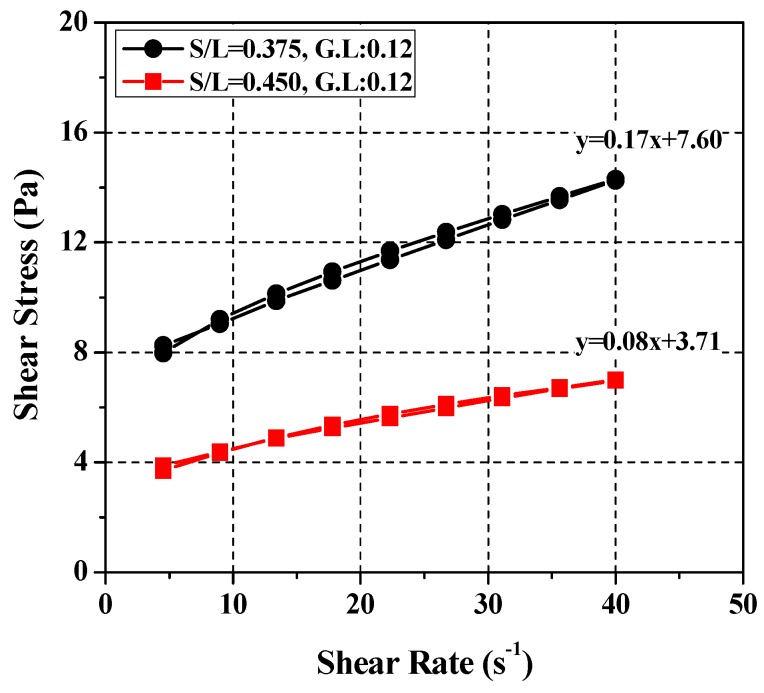
Test results of rheology for SRM.

To validate the equation expressing the relationship between the cement paste and SRM mixes, the values obtained were compared with the corresponding measurements under various *W*/*C* conditions. After excluding cement paste mixes lower than *W*/*C* = 0.37 in Table 6, which exceeds the maximum torque capacity of the rheometer, rheological analysis was performed on two mixes exceeding *W*/*C* = 0.45, i.e., *W*/*C* = 0.50 and 0.55 (see Table 7). Applying Equation (1), the SRM mixing ratios corresponding to the cement paste mixing ratios were calculated at *W*/*L* = 0.375 and *G*/*L* = 0.12 for *W*/*C* = 0.50 and *W*/*L* = 0.45 and *G*/*L* = 0.12 for *W*/*C* = 0.55. Figure 10 shows the results of rheological analysis of the two cement paste mixes (*W*/*C* = 0.50 and 0.55). Each analysis was based on multiple experimental runs at a constant temperature and a constant time. The results of the rheological analysis of each SRM mix using the equation are plotted in Figure 11. The comparison and analysis results show that the SRM mixing ratios were derived using Equation (2) and the results simulated the cement paste rheological properties more accurately. This suggests that the cement paste and SRM mixing ratios derived in this study are valid. In the present study, the test capacity of the rheometer was greater under 0.37 or less and it was difficult to analyze the rheological properties due to the low viscosity under the condition of *W*/*C* = 0.55 or more. Therefore, in Equation (2), the *W*/*C* ratio is validated in the range of 0.37~0.55.

## 5. Conclusions

This study examined the mixing ratios of SRMs capable of simulating the rheological properties of different cement paste mixes by drawing on the results of a previous study on the material composition of SRMs for cement paste, which can become the most fundamental standard and the first step toward developing SRMs for concrete. Based on various experimental results, it could suggest that SRM mixing ratios can reliably simulate cement paste rheological properties with different *W*/*C*. The key study results are summarized here.

First, the reference ranges of the yield stress and plastic viscosity of the selected mixing ratios were derived by analyzing the rheological properties of cement paste at each ratio. As a result, the plastic viscosity and yield stress decreased when the *W*/*C* ratio increased. To present the SRM mixing ratios satisfying the reference ranges derived, the influence of each SRM component on the overall rheological properties was investigated. As a result, the yield stress was analyzed and found to be affected by the amount of limestone under the constant water:glycerol ratio. In addition, plastic viscosity is analyzed to be affected by the ratios of water:glycerol under a constant *S*/*L* ratio. At the same time, the minimum glycerol content to prevent material separation and hysteresis behavior was 12% of the limestone content. The results show that all the mixing ratios satisfied the reference ranges of rheological parameters for all cement paste mixes tested.

Rheological analysis of the SRM mixing ratios showed that their rheological performance satisfied all SRM requirements under all conditions. An equation expressing the relationship between the cement paste and SRM was suggested and was used to derive the SRM mixing ratios for their respective cement paste mixes. The equation was validated by using *W*/*C* dependent rheological analysis of various cement paste mixes. All of the cement past mixes verified the proposed relationship between the cement paste and the suggested SRM mixing ratio.

## Figures and Tables

**Table 1:** Analysis of limestone components.

Item	Particle Size	Density	Constituent (%)				
	(μm)	(g/cm^3^)	SiO_2_	Al_2_O_3_	Fe_2_O_3_	MgO	CaO
Limestone	20	2.7	0.3	0.1	0.16	0.2	99.3

**Table 2:** Analysis of glycerol components.

Item	Constituent (%)								
	Content	NH_4_	SO_4_	As	Fe	Pb	Acid-Base	Fatty Acid Ester	Sulfate
Glycerol	99	0.005	0.002	0.0002	0.0003	0.0004	0.005	0.2	0.015

**Table 3:** Measured rheological ranges for tested mixing ratios.

Mixing Ratio	Plastic Viscosity (Pa·s)	Yield Stress (Pa)
W/C = 0.37	1.0 ± 0.1	22.0 ± 3.0
W/C = 0.40	0.5 ± 0.1	15.0 ± 3.0
W/C = 0.45	0.3 ± 0.1	10.0 ± 3.0

**Table 4:** Comparison of flow characteristics between cement pastes and suggested standard reference materials.

Items	Cement Paste			Standard Reference Materials		
Mixing Ratios	*W*/*C* = 0.37	*W*/*C* = 0.40	*W*/*C* = 0.45	*W*/*L* = 0.27	*W*/*L* = 0.31	*W*/*L* = 0.33
				*G*/*L* = 0.12		
Plastic viscosity (Pa·s)	1.0 ± 0.1	0.5 ± 0.1	0.3 ± 0.1	1.1	0.5	0.3
Yield stress (Pa)	22.0 ± 3.0	15.0 ± 3.0	10.0 ± 3.0	19.1	15	10.6

**Table 5:** Review of standard reference material requirements.

Items			Requirements				
No	*W*/*L*	*G*/*L*	Separating Resistance	Linearity	Yield Value	Hysteresis	Chemical Stability
1	0.27	0.12	O ^1^	O	O	O	O
2	0.31		O	O	O	O	O
3	0.33		O	O	O	O	O

^1^ O indicates conformity.

**Table 6:** Comparison between SRM mixing ratios and cement paste mixing ratios.

No.	*W*/*L*	*G*/*L*	*W*/*C*
1	0.27	0.12	0.37
2	0.31		0.40
3	0.33		0.45

**Table 7:** Mixing ratios of cement paste and SRM for verification.

No.	*W*/*C*	*W*/*L*	*G*/*L*
1	0.50	0.375	0.12
2	0.55	0.45	
